# Toward the Beginning of Time: Circadian Rhythms in Metabolism Precede Rhythms in Clock Gene Expression in Mouse Embryonic Stem Cells

**DOI:** 10.1371/journal.pone.0049555

**Published:** 2012-11-14

**Authors:** Jiffin K. Paulose, Edmund B. Rucker, Vincent M. Cassone

**Affiliations:** 1 Department of Biology, Center for Research on Biological Clocks, Texas A&M University, College Station, Texas, United States of America; 2 Department of Biology, University of Kentucky, Lexington, Kentucky, United States of America; Vanderbilt University, United States of America

## Abstract

The appearance, progression, and potential role for circadian rhythms during early development have previously focused mainly on the suprachiasmatic nucleus (SCN) and peri- and postnatal expression of canonical clock genes. More recently, gene expression studies in embryonic stem cells have shown that some clock genes are expressed in undifferentiated cells; however rhythmicity was only established when cells are directed toward a neural fate. These studies also concluded that a functional clock is not present in ESCs, based solely on their gene expression. The *null* hypothesis underlying the present study is that embryonic stem cells become rhythmic in both clock gene expression and glucose utilization only when allowed to spontaneously differentiate. Undifferentiated stem cells (ESCs, n = 6 cultures/timepoint for all experiments) were either maintained in their pluripotent state or released into differentiation (dESCs, n = 6 cultures/timepoint for all experiments). Glucose utilization was assayed through 2-deoxyglucose uptake measurement, and clock gene and glucose transporter expression was assayed every 4 hours for 2 days in ESCs and dESCs by quantitative PCR (qPCR) in the same cell lysates. Undifferentiated stem cells expressed a self-sustained rhythm in glucose uptake that was not coincident with rhythmic expression of clock genes. This physiological rhythm was paralleled by glucose transporter mRNA expression. Upon differentiation, circadian patterns of some but not all clock genes were expressed, and the amplitude of the glucose utilization rhythm was enhanced in dESCs. These data provide the earliest evidence of a functional circadian clock, in addition to further challenging the idea that rhythmic transcription of clock genes are necessary for rhythmic physiological output and suggest a role for a clock-controlled physiology in the earliest stages of development.

## Introduction

Circadian rhythms and the cellular/physiological processes they control have been established as a highly conserved mechanism by which organisms are able to anticipate daily changes in the environment and to temporally coordinate complex processes [1]. These time-keeping mechanisms have been observed in nearly all taxa and, while there is variability in the details of circadian organization at the cellular level, all possess similar formal characteristics such as endogenous generation under constant conditions, temperature compensation and synchronization with environmental cues, or *Zeitgebers*, such as light or timed feeding [2].

In mammals, circadian rhythmicity is maintained via a transcriptional/translational feedback loop in which positive elements *mClock* and *mBmal1* heterodimerize to activate transcription of negative elements Periods (*mPer1, 2,* and *3*) and Cryptochromes (*mCry1* and *2*) via binding to E-box elements within the promoter regions of those negative elements. PER and CRY proteins heterodimerize and reenter the nucleus to inhibit the interaction of positive elements CLOCK and BMAL1. Thus, the PER/CRY inhibition itself acts to repress these genes’ own transcription, which is eventually relieved by Casein Kinase-1 Epsilon-mediated proteosomal degradation [2]. Additionally, the nuclear receptors REVERB-A and ROR-a competitively bind retinoic acid-related orphan receptor response elements (RORE) on the *mBmal1* promoter region and act to inhibit and activate *mBmal1* transcription, respectively [3]. These nuclear receptors have been suggested as a crucial link between the circadian timekeeping mechanism and physiology [4]. Together, this transcriptional/translational feedback loop is frequently referred to as the “canonical molecular circadian clock” [2].

At the systems level, the master pacemaker that confers time of day information throughout the organism resides in the suprachiasmatic nucleus (SCN). This paired structure - which is located in the anterior hypothalamus and situated just above the optic chiasm – consists of 20,000 neurons and receives photic input from the retina. Lesion of the SCN abolishes physiological and behavioral rhythms, demonstrating its role in conferring time of day information to coordinated behaviors and physiological processes [5]–[7]. Furthermore, transplantation of SCN tissues [8], [9] or cells [10] restores rhythmicity such that the recipient expresses the period and phase of the donor tissue, confirming that the SCN is both necessary and sufficient as a circadian pacemaker.

Previously, investigations into the development of the mammalian circadian system have concentrated primarily on the development of the SCN pacemaker [11] or, more recently, on the ontogeny of rhythms in peripheral tissues [12], [13]. These studies showed that the onset of molecular circadian rhythms in the brain occurs just after birth, followed by rhythms expressed by peripheral tissues. However, the postnatal rhythms of clock gene expression and electrical activity in the SCN are preceded by rhythmic glucose uptake 3–4 days before parturition and well before the completion of synaptogenesis within the SCN [14].

Several studies have investigated clock gene expression earlier in mammalian embryogenesis. Saxena et al [15] investigated embryonic *mPer1* expression *in utero* using live fluorescence imaging. Although this was a proof of concept study, the authors clearly showed *mPer1::luciferase* expression as early as day 7. Earlier, Johnson et al. [16] showed evidence of zygotic expression of several clock genes in the pre-implantation embryo as well as uterine tissues, suggesting that perhaps clock gene expression plays a role in embryo-uterus interaction during early embryogenesis. It is important to note, however, that these studies were from embryos taken directly from the uterus, placing the expression of embryonic genes in the context of the uterine environment.

Recently, two independent groups have investigated circadian rhythms in embryonic stem cells. Yagita et al. [17] used ES cells stably transfected with bioluminescent luciferase driven by either an *mBmal1* promoter or clock-controlled gene *mDbp* promoter. The results from this study showed that undifferentiated cells were not rhythmic with respect to *mBmal1* or *mDbp,* but cells that were directed towards a neural fate were rhythmic after synchronization with forskolin. Kowalska et al. [18] expanded on those experiments and showed that individual undifferentiated ESCs are not rhythmic. Both of these studies provided ample evidence that the canonical molecular clock is not rhythmic in ESCs and concluded that the clock is not functional this early in development. However, given the previous evidence that glucose uptake – a well-established physiological output of the circadian clock - is rhythmic in the SCN before clock genes are rhythmic during development, it is premature to equate lack of canonical clock gene rhythmicity to a lack of a functional timekeeping mechanism.

Here we present evidence that rhythmicity in ESCs precedes the development of clock gene rhythms. Primary ESCs were cultured either in the presence or absence of the differentiation inhibitor Leukemia Inhibitory Factor (LIF). 2-DG uptake was assayed as a physiologically relevant output of the clock and a comprehensive profile of transcripts from both positive and negative limbs of the molecular clock was analyzed for rhythmicity over 2 days in culture, as well as the “stabilizing loop” consisting of *mReverb-a* and *mRor-a*.

## Materials and Methods

### Cell Culture

The ES cells were derived from blastocysts of SV129 mice for molecular studies and C57BL/6 mice for the real-time analyses. Females (3 weeks of age, n = 3–5) were superovulated by i.p. injection of pregnant mare serum gonadotropin (PMSG, 5IU, Sigma, St. Louis, MO) followed by human chorionic gonadotropin (hCG, 2.5IU, Sigma, St. Louis, MO) prior to mating with males of the same respective genotype). ES colonies were initially expanded from a 96-well format to 24-well plates, and finally to 6-well plates while being maintained on mitomycin C-inactivated STO feeder cells. For the experiments, ES cells were passaged nine times into feeder-free cultures, resulting in pure ESC cultures that were maintained as pluripotent ESCs or allowed to differentiate by the removal of LIF, which was done one passage prior to experimentation (passage 8). The culture environment consisted of a standard water-jacketed incubator held at 37°C and 5% CO2 and controlled humidity. Standard ES media (ES-DMEM), Dulbecco’s Modified Eagle Medium (DMEM) supplemented with 15% fetal bovine serum, sodium bicarbonate (2.2 g/L), MEM Non-Essential Amino Acids (0.1 mM), L-glutamine (2 mM), B-mercaptoethanol (0.1 mM), penicillin (50 U/ml)/streptomycin (50 µg/ml) antibiotic (1 mM), and LIF (500–1000 U/ml, Millipore, MA) was used for maintenance of ES cell pluripotency. Differentiation medium was identical to ES-DMEM but without LIF. Cultures were maintained in 100 mm, gelatin-coated culture dishes, fed daily, passaged every 2 days and replated at 1×10^6^ cells/well into 6-well culture plates (BD Bioscience) for experiments. Cells were allowed to grow for 12 hours after final passage before sampling began, at which point time is referred to as 0 hours on each graph.

### ^14^C-2-DG Uptake Assay

At the onset of each timepoint, one 6-well plate from each cell type was incubated with ^14^C-2-deoxyglucose (2-DG, 0.1 mCi/ml; American Radiolabeled Chemicals, St. Louis, MO) for one hour by complete removal and replacement of the medium. The medium was then removed and the cells rinsed twice with Dulbecco’s PBS (Invitrogen). Cells were harvested in 1 ml Trizol reagent (Invitrogen) to extract cellular RNA and soluble protein. 200 µl of cell lysate was placed in 5 ml of scintillant and each sample counted in duplicate on a Beckman scintillation counter. Disintegrations per minute (DPM) were converted to molar quantities as per Sokoloff’s method [19]. 2-DG uptake was normalized to total RNA as measured by spectrophotometry on a Nanospec 1000 (Nanometrics, Milpitas, CA). Total RNA was isolated from the remaining cell lysate and subsequently treated with DNase I (Invitrogen) for quantitative PCR analysis of gene expression.

### Real-Time Quantitative Polymerase Chain Reaction

DNase-treated cDNA from total RNA was generated using Superscript II reverse transcriptase (Invitrogen). Relative quantification of clock genes and stem cell marker genes was accomplished using SYBR chemistry-based qPCR on either ABI Prism 7500 Fast or StepOne Plus system (Applied Biosystems Foster, CA). Both machines are capable of generating identical thermal profiles and this was confirmed by testing identical samples on both machines. Table S1 lists all genes profiled and the corresponding primer sequences which were identified through literature search or using Primer Express software (Applied Biosystems). The relative quantification was based on a standard curve of dilutions 1∶50, 1∶100, 1∶250, 1∶500, and 1∶1000 with triplicate samples diluted 1∶100. All transcripts were normalized to corresponding values of *mCyclophilin D*.

### Real-Time Bioluminescent Protein Expression

ESCs of C57Bl/6/SV129 hybrid mice expressing the PER2::LUCIFERASE fusion protein (Yoo, et al., 2004) were used to monitor the real-time expression of mPER2 throughout differentiation. For these studies, feeder-free ESCs or dESCs (passage 9) were plated onto gelatin-coated 35 mm dishes (n = 10 for both ESCs and dESCs) (BD-Falcon) in ES media at 1×10^5^ cells per plate and allowed to attach and grow for 12–15 hours. At the onset of the experiment, media was replaced with ES media with the following modifications: Sodium bicarbonate concentration reduced to 0.35 g/L and 10 mM HEPES added to compensate for atmospheric conditions. The dishes were sealed with glass coverslips and vacuum grease and placed in a Lumicycle luminometer (Actimetrics, IL). One group of cells was maintained in the undifferentiated state via LIF administration, while another group of cells, passaged from the same initial culture, did not receive LIF and, therefore, underwent differentiation. Bioluminescence recording and detrended plotting of the counts from the cells was accomplished using a Lumicycle photomultiplier detection system and Lumicycle software, respectively (Actimetrics, IL).

### Statistics

Time course data were subjected to cosinor analysis based on linear harmonic regression (Circwave Software, Roelof Hut) where each 24-hour period was tested separately. The software assumes a 24 hour period and applies harmonics to the basic sinusoidal function. These attributes allowed us to use the software to determine whether the data were rhythmic for each day of the 2 day sampling as well as providing centers of gravity to determine acrophase for each day. The criteria for determining *bona fide* circadian rhythms were as follows: a) both days show statistically significant rhythms using Circwave analysis and b) the acrophases of day 1 and day 2 occur within 23–25 hours of each other. Data were also tested using ANOVA for non-rhythmic expression profiles to determine significant changes over time. ANOVA was performed using Sigmastat software (Systat Software Inc., Point Richmond, CA). Where performed, amplitudes were calculated by subtracting absolute peak levels from previous or following trough levels - as previously determined using ANOVA - and dividing by 2.

## Results

### Stem Cell Morphology/identity

**Figure 1:**
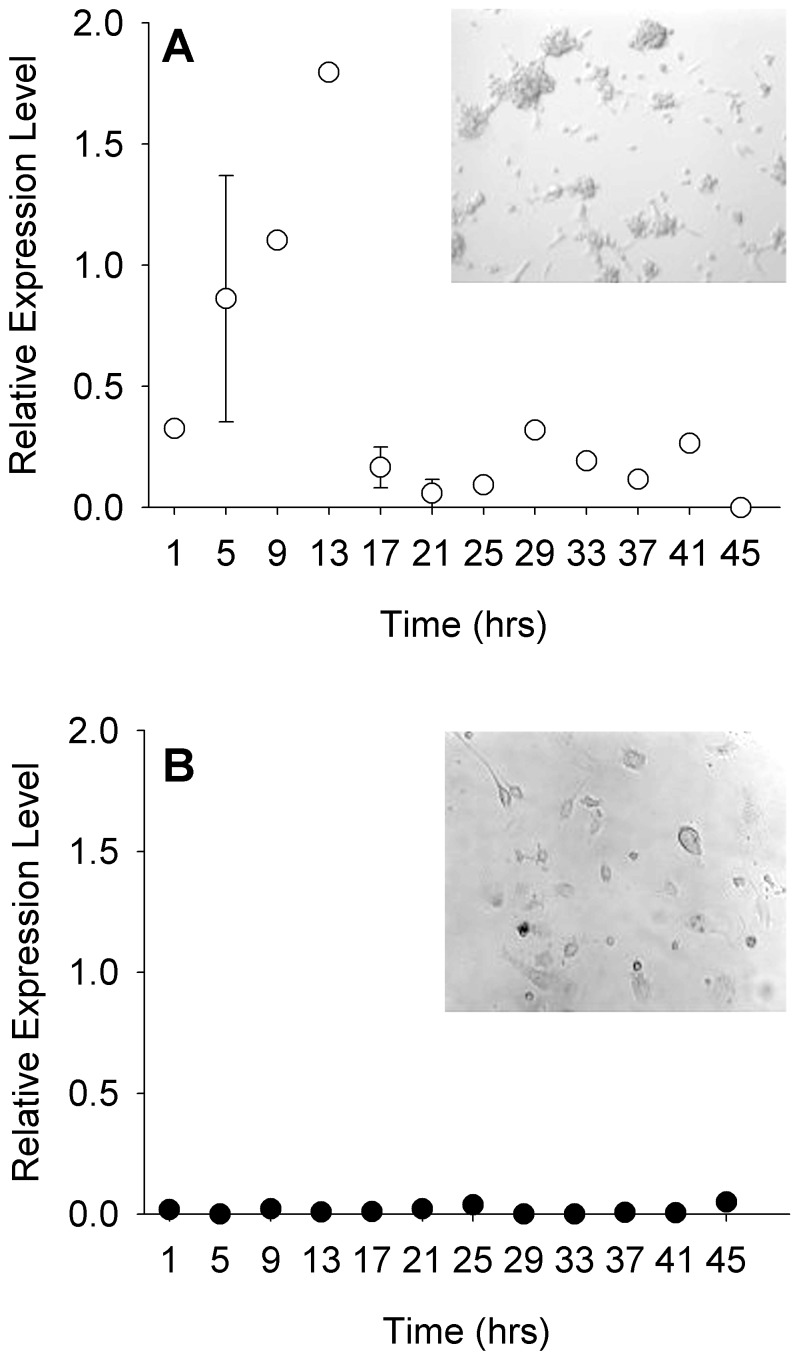
ESCs and dESCs are distinguishable by morphology and *mOct4* expression. Relative expression levels of *mOct4* and photomicrographs of ESCs (A, n = 6 and A inset, respectively) and dESCs (B, n = 6 and B inset). Error bars indicate ± SEM.

ESCs maintained under LIF displayed morphology consistent with that of undifferentiated embryonic stem cells when imaged just before sampling (Fig. 1A). Multiple cells formed isolated colonies in which individual cell borders were indistinct and colony edges rounded. Flattened cells, indicative of differentiation are minimal across cultures. Additionally, *mOct4* expression was high during the first day of culture and decreased during the second, suggesting that some of the cells began differentiating on the second day (Fig. 1A, inset). Acutely differentiated cells, also imaged just before sampling began, were morphologically distinct from undifferentiated cultures. Individual cells were easily identifiable and flattened in appearance (Fig. 1B). Furthermore, *mOct4* expression was very low across both days of sampling, confirming that the cultures were differentiated (Fig. 1B, inset).

### 2-DG Uptake

**Figure 2:**
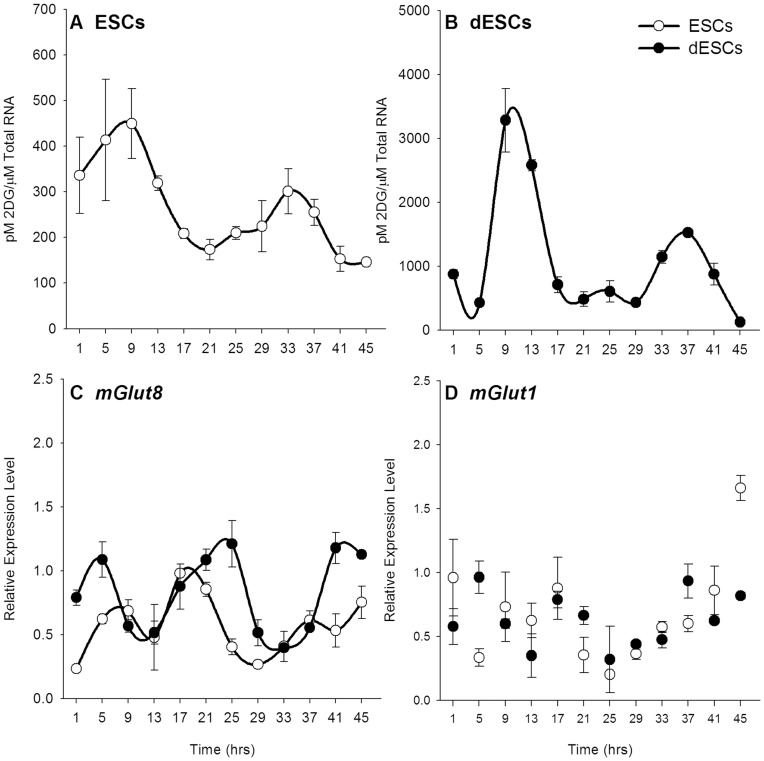
2-DG uptake and glucose transporter 8 expression are rhythmic in ESCs and dESCs. 2-DG uptake as normalized to total RNA levels in ESCs (A, n = 6) and dESCs (B, n = 6). C) *mGlut8* mRNA expression is rhythmic in both ESCS (n = 6, white circles) and dESCs (n = 6, black circles). D) *mGlut1* expression was not rhythmic in either culture. Lines connecting scatter denote statistically rhythmic oscillations. Note that the ordinate axis is different in A than in B to better illustrate the low-amplitude rhythm in undifferentiated cells. Error bars indicate ± SEM.

ESCs cultured in the presence of LIF exhibited two-fold oscillations of 2-DG uptake over two cycles, with peaks occurring at 9 and 33 hours of sampling (p<0.001, Fig. 2A). Acutely differentiated cells were similarly rhythmic with peak uptake occurring at approximately 9 and 37 hours (p<0.001, Fig. 2B). There was no significant difference in acrophase between ESCs and dESCs. Furthermore, the amplitude of 2-DG uptake in dESCs was markedly increased, as was the basal level of uptake, each being almost 10-fold higher than the corresponding level in undifferentiated ESCs (p<0.001).

### Glucose Transporter Gene Expression

The rhythms in 2-DG uptake suggested that glucose transporter (*mGlut*) expression might also be rhythmic. qPCR analysis of 6 different *mGlut* members revealed only two that were detectable: *mGlut1* and *mGlut8*. Of these two, *mGlut8* was rhythmic in both ESCs and dESCs (p<0.001, Fig. 2C). In both cell types, the expression profile of *mGlut8* was phase delayed to that of the 2-DG uptake; the rhythms peaked between 17 and 21 hours and again towards the end of the sampling period, around 45 hours. There was no statistically significant difference in overall expression levels of *mGlut8* between either cell types. *mGlut1* was not rhythmic in either ESCs or dESCs (Fig. 2D).

### Clock Gene Expression

**Figure 3:**
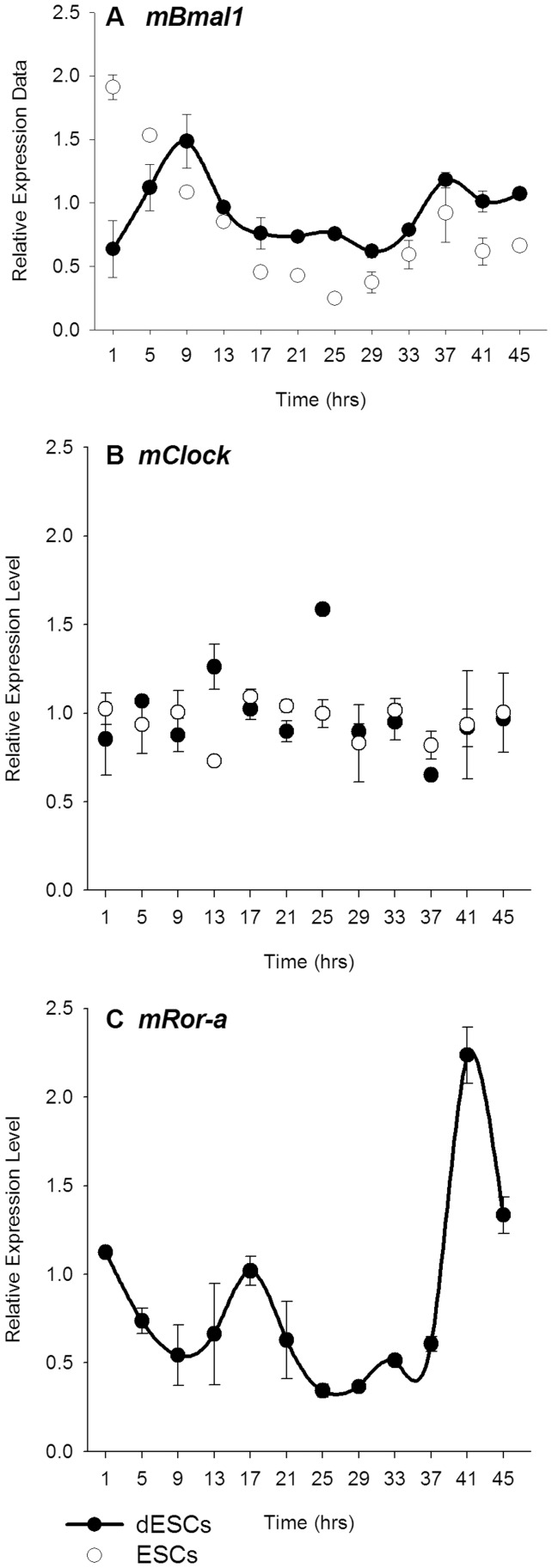
Positive elements of the molecular clock are arrhythmic or not detectable in ESCs, but some are rhythmic in dESCs. qPCR data comparing relative clock gene mRNA expression in ESCs (n = 6, white circles) and dESCs (n = 6, black circles). Comparisons of A) *mClock*, B) *mBmal1* and, C) *mRor-a*. Line segments connecting data points denote statistically rhythmic oscillations. Error bars indicate ± SEM.

**Figure 4:**
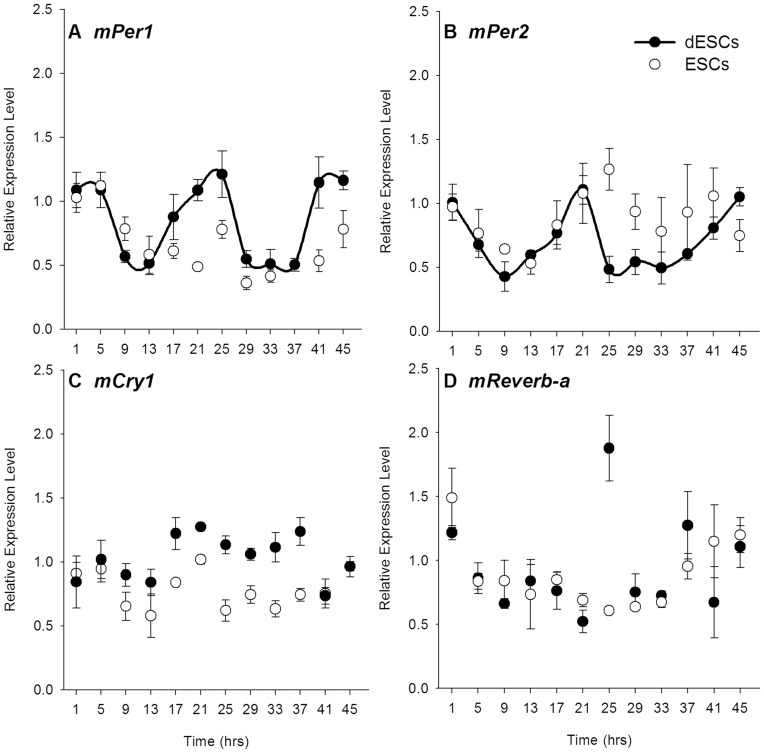
Negative elements of the molecular clock are arrhythmic in ESCs, but most are rhythmic in dESCs. Relative expression levels of A) *mPer1*, B) *mPer2*, C) *mCry1*, and D) *mReverb-a* mRNA in ESCs (n = 6, white circles) and dESCs (n = 6, black circles). Line segments connecting data points denote statistically rhythmic oscillations. Error bars indicate ± SEM.

**Figure 5:**
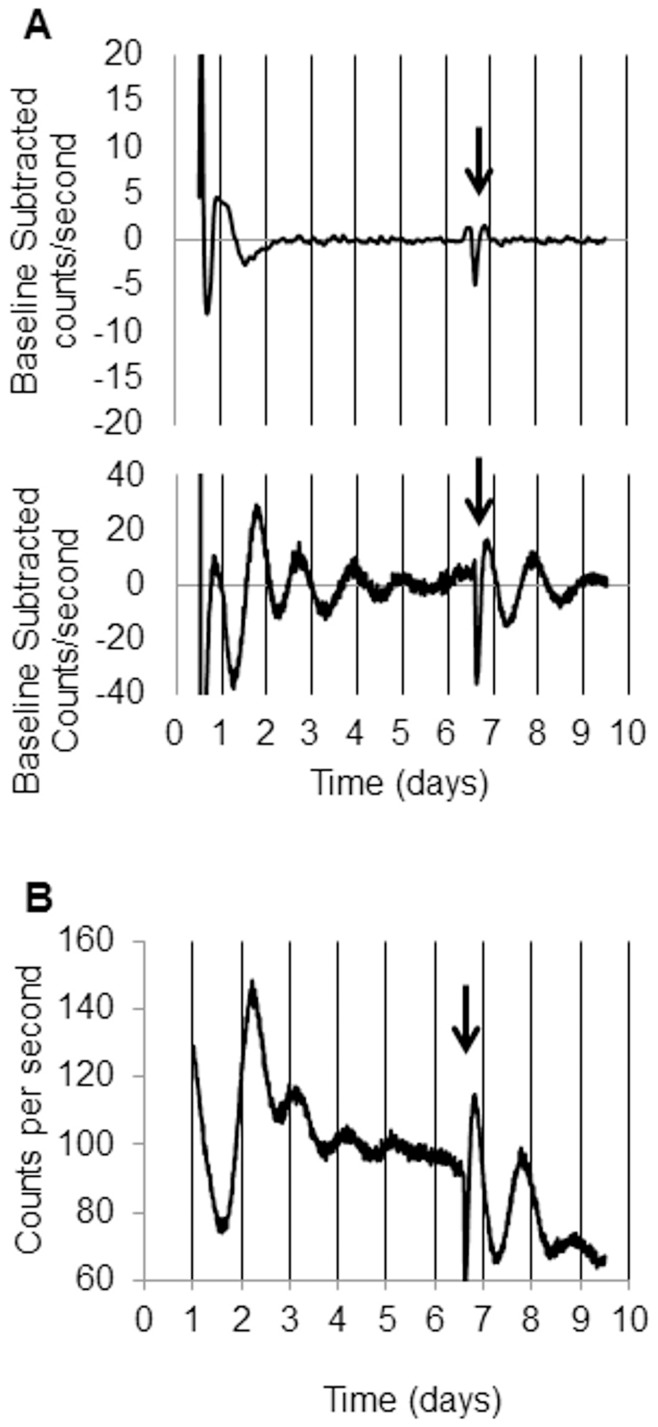
mPER2 protein is arrhythmic in ESCs and rhythmic in dESCs. Baseline subtracted bioluminescence of mPER2::LUCIFERASE in A, Top Panel) ESCs and A, Bottom Panel) dESCs. B) Raw data of dESC bioluminescence. Arrows indicate media exchange.

Rhythmic 2-DG uptake and glucose transporter expression in both ESCs and dESCs suggested that the canonical molecular clockwork may be present in these cultures. However, quantitative, real-time PCR against clock gene transcripts revealed differential expression patterns both within and between the two conditions. The positive elements, *mClock* and *mBmal1* were not rhythmic in ESCs (white circles, Fig. 3A and B, respectively). Upon differentiation, however, only *mBmal*1 displayed circadian rhythmicity (p<0.05, Fig. 4B, black circles). *mRor-a* was not detectable in ESCs, but was rhythmic in dESCs, peaking at approximately 17 hours (p<0.001, Fig. 3C). Among the negative elements, neither *mPer1* nor *mPer2* transcripts were rhythmic in ESCs, but the temporal profiles of both were similar (Fig. 4A and B, respectively, white circles). Both *mPer1* and *mPer2* were rhythmic in dESCs, however, with peaks occurring at 21 hours of sampling in both (p<0.001, Fig. 4A and B, respectively, black circles. Neither *mCry1* nor *mReverb-a* were rhythmic in ESCs or dESCs (Fig. 4C and D, respectively). The expression pattern of mPER2 protein, as visualized by real-time bioluminescence, was similarly non-rhythmic in ESCs, but highly rhythmic in dESCs (p<0.001, Fig. 5A and B, respectively). Furthermore, the rhythm in dESCs was reinstated by culture medium exchange (Fig. 5B, arrow) despite a diminishing baseline of protein expression which may be attributed to cell death over time.

## Discussion

Rhythmic glucose utilization precedes the development of the molecular clockworks in embryonic stem cells; however clock gene expression becomes rhythmic upon short-term differentiation. Previous investigations of the ontogeny of circadian rhythms concluded that undifferentiated mouse embryonic stem cells do not contain a functional clock based upon the lack of molecular rhythmicity in the expression of known components of the molecular clock [2], both in synchronized cultures as well as at the single cell level [17], [18]. These studies, however, limited their analyses to some, but not all, putative clock genes. Here we show – in agreement with those studies as well as in a more comprehensive manner – that undifferentiated cells indeed do not possess a functioning canonical molecular clock, based upon expression of mRNA of most of these genes as well and protein expression of PER2. Undifferentiated cells were not rhythmically expressed with respect to the clock genes tested, which is also in accord with previous studies. Upon non-specific differentiation, however, all of the tested clock genes were expressed rhythmically, with the exception of *mClock*, *mCry1,* and *mReverb-a*. Furthermore, the rhythm of luciferase bioluminescence in mPER2::LUC dESCs confirms the expression data. Although previous studies also looked at differentiated cells, the fate of those cultures was directed towards that of neural tissues. These data show the earliest developmental point at which clock genes exhibit circadian rhythms. This study investigated gene expression rhythms in primary cell cultures without the use of chemical synchronization as well as a physiological output of the clock, glucose uptake, which is a measure directly indicative of glucose utilization [19]. In addition to investigating whether undifferentiated cells exhibit uninduced rhythmicity, undirected differentiation was included in this set of experiments in order to observe any potential reorganization of clock elements in a manner that recapitulates the development of the embryo *in utero.*

Remarkably, rhythmic glucose utilization in undifferentiated stem cells does not necessarily coincide with rhythmic canonical clock gene expression; these processes are developmentally and experimentally separable. Previous studies in juvenile chicks showed that enucleation abolishes 2-DG uptake in the brain while clock genes remained rhythmic [20]. Similarly, rhythmic administration of melatonin to embryonic astrocytes was sufficient to drive rhythms of 2-DG uptake, but not of all the canonical clock genes [21]. Along with the previously mentioned rhythm of 2-DG uptake in neonatal rats [14] as well as the recently discovered transcription-independent rhythm of redox cycles in human red blood cells [22] the data presented here provide compelling evidence that metabolic rhythms are not regulated solely by the canonical molecular clockworks.

The glucose utilization rhythms in both ESCs and dESCs were corroborated by rhythmic glucose transporter expression, *mGlut8* in both ESCs and dESCs, indicating that the rhythms are driven by a transcriptional mechanism separate from the rhythmic expression of the canonical clock genes. Previously, Tonack, et al. [23] showed that ESCs expressed several *mGlut* transcripts throughout embryoid body differentiation, including glut1 and glut8 in undifferentiated cultures. Although neither *mGlut1* nor *mGlut8* have been implicated in circadian rhythms, *mGlut1* is necessary for ESC viability [24] and upregulation of *mGlut8* in embryoid bodies suggests an increased need for glucose in differentiating cells [23]. Interestingly, the amplitude of *mGlut8* in both cultures remained the same, while the average glucose utilization was markedly increased in differentiated cells.

The rhythmic clock gene expression upon differentiation is quite remarkable for a number of reasons. The relative profiles of those genes that were rhythmic are consistent with the canonical molecular mechanism of circadian transcription, the negative elements *mPer1* and *mPer2* were identical in their phasing, and the positive element *mBmal1* was expressed in anti-phase. Also, the cultures were rhythmic in the absence of any chemical synchronization. Previous studies had used dexamethasone [17], [18] or forskolin [17] to synchronize cultures, as is common practice. In this study, the only conceivable synchronizing factors could have been centrifugation during passage or the absence of LIF, however there is no evidence of either phenomenon occurring in ESCs. Despite this, rhythms in 2-DG and clock genes were synchronized differentially between ESCs and dESCs. Secondly, the signal for differentiation away from the pluripotent state immediately synchronized the cultures in this study, as seen in the bioluminescence data. In contrast, Yagita et al [17] showed that clock genes remained arrhythmic throughout an induced differentiation process that required several days of incubation with retinoic acid. In combination with the data presented here, it would appear that clock gene rhythmicity is a dynamic phenomenon that can change depending on developmental stage. Indeed, previous studies have shown that the synchrony of clock genes changes throughout development in both the SCN [13] and the liver [12]. The nature of this synchronization is unknown but, considering the aggregate nature of the cultures, functional gap junctions between cells may facilitate communication and synchrony [25]. Finally, the relative phases of the clock genes, once synchronized, align in a manner that would suggest functional molecular clockworks, with the positive and negative elements expressed anti-phase to each other.

Unlike previous studies examining rhythms in fate-specific differentiated cells, the media in this study differed with respect to one ingredient; the differentiation inhibitor LIF. LIF acts as a cytokine binding to a heterodimeric receptor complex of its own receptor, LIFR, and gp130. The pathway ultimately leads to activation and translocation of STAT3 to the nucleus where it binds and activates various genes, presumably those involved in maintaining pluripotency [26]. However, there is no known, direct link between the LIF pathway and glucose transporter/uptake. The absence of LIF in differentiated cultures may account for the rhythmic expression of clock genes, but it does not explain the persistence of the 2-DG uptake rhythm in undifferentiated cells.

These data cannot be explained by possible effects of 2-DG on metabolism itself. While high mM concentrations 2-DG inhibits cellular metabolism *in vitro* [27], and high dosages of 2-DG can affect circadian clock light sensitivity *in vivo* [28], the concentrations of ^14^C-2DG employed here (8 µM) and elsewhere *in vitro* [10], [21], and dosages employed for metabolic markers *in vivo* have no effect on metabolism [19], [29] or circadian clock function [30]. Further, the 2-DG concentrations employed here are 1/250 of the 2 mM Kondoh et al. [31] have shown to have no effect on ES cell proliferation. Much higher concentrations than 2 mM are required to affect differentiation. Moreover, these data cannot be explained by cell cycle effects on clock gene expression and/or metabolism, since cell-cycle period for these cells is 11–12 hours rather than the 24 hours observed here [32]. The most parsimonious explanation of the present results is that a circadian clockworks that does not entail the rhythmic expression of clock genes is present in mouse embryonic stem cells before differentiation into germ lines.

Thus, although undifferentiated ESCs do not possess a functioning canonical molecular clock, a circadian rhythm of glucose utilization persists in these cells. This rhythm is coincident with rhythmic expression of one glucose transporter gene, suggesting rhythmic transcriptional control of glucose utilization. Whether this rhythm is initiated intrinsically or in response to glucose availability is unknown. In addition, acute differentiation by withdrawal of LIF increases the amplitude of glucose utilization rhythms and initiates rhythms of the expression of canonical clock genes, suggesting a potential role for the cytokine in initiating the transcriptional-translational feedback loop.

## Supporting Information

Table S1 Primers used for qPCR analysis.
(DOCX)
