# Supplementary material for: Toward the Beginning of Time: Circadian Rhythms in Metabolism Precede Rhythms in Clock Gene Expression in Mouse Embryonic Stem Cells
Source: PLoS One. 2012 Nov 14;7(11):e49555. doi: 10.1371/journal.pone.0049555 (PMC3498230; doi:10.1371/journal.pone.0049555)
Supplement: Table S1 — Primers used for qPCR analysis. (DOCX) [file pone.0049555.s001.docx]

| Gene | Forward Primer Seq. 5’-3’ | Reverse Primer Seq. 5’-3’ |
| --- | --- | --- |
| *mCycD* | CTCATCTGGACGGGAAAAT | CCAGTCATCCCCTTCTTTCA |
| *mGlut8* | GCTCTCAGTGTCCTATTCA | AAATGGGCTGTGACTTGT |
| *mGlut1* | CAGGAGGATATTCAGGACTT | CAGTGTGGAGATAGGAGAG |
| *mClock* | GGCGTTGTTGATTGGACTAGG | GAATGGAGTCTCCAACACCCA |
| *mBmal1* | GGACTTCGCCTCTACCTGTTCA | AACCATGTGCGAGTGCAGGCGC |
| *mRor-a* | CCAACCGTGTCCATGGCAGAAC | GCACACAGCTGCACATCACCT |
| *mPer1* | GCTGGGCCGGTTTTGTG | CACTTTATGGCGACCCAAC |
| *mPer2* | ATTGGGAGGCACAAAGTCAG | ATCAGTAGCCGGTGGATTTG |
| *mCry1* | TACAGCAGCCACAAACAACC | TTCTTGTCCCAAGGGATCTG |
| *mReverb-a* | CCCTGGACTCCAATAACAACACA | GCCATTGGAGCTGTCACTGTAG |
